# Salt and Sugar: Two Enemies of Healthy Blood Pressure in Children

**DOI:** 10.3390/nu13020697

**Published:** 2021-02-22

**Authors:** Simonetta Genovesi, Marco Giussani, Antonina Orlando, Francesca Orgiu, Gianfranco Parati

**Affiliations:** 1 School of Medicine and Surgery, University of Milano-Bicocca, 20100 Milan, Italy; francesca.orgiu@unimib.it (F.O.); gianfranco.parati@unimib.it (G.P.); 2 Istituto Auxologico Italiano, IRCCS, Cardiology Unit, 20100 Milan, Italy; antonina.orlando@unimib.it; 3 Family Pediatrician, Agenzia Tutela Salute, 20100 Milan, Italy; abrjg@tin.it

**Keywords:** children, hypertension, obesity, salt, sodium, fructose, uric acid

## Abstract

The prevalence of essential arterial hypertension in children and adolescents has grown considerably in the last few decades, making this disease a major clinical problem in the pediatric age. The pathogenesis of arterial hypertension is multifactorial, with one of the components being represented by incorrect eating habits. In particular, excessive salt and sugar intake can contribute to the onset of hypertension in children, particularly in subjects with excess weight. Babies have an innate predisposition for sweet taste, while that for salty taste manifests after a few weeks. The recent modification of dietary styles and the current very wide availability of salt and sugar has led to an exponential increase in the consumption of these two nutrients. The dietary intake of salt and sugar in children is in fact much higher than that recommended by health agencies. The purpose of this review is to explore the mechanisms via which an excessive dietary intake of salt and sugar can contribute to the onset of arterial hypertension in children and to show the most important clinical studies that demonstrate the association between these two nutrients and arterial hypertension in pediatric age. Correct eating habits are essential for the prevention and nondrug treatment of essential hypertension in children and adolescents.

## 1. Introduction

In ancestral times, salt and sugar were difficult to find, but were of important nutritional value for our forefathers. This has generated a marked preference for sweet and salty taste in our species. Babies have an innate predisposition for sweet taste, while that for salty taste becomes evident after a few weeks [1]. Moreover, salt and sugar favor the preservation of food, and this has led to the development of numerous recipes and food preparations that involve their use, such as cheeses, salami, and jams. The recent modification of the styles of diet and the current very wide availability of salt and sugar have led to an exponential increase in the consumption of these two nutrients. In adults today, the consumption of salt is about double compared to what is recommended by the World Health Organization (WHO) [2], while the per capita consumption of sugar in the Anglo-Saxon population has gone from a few kg/year at the beginning of the last century to more than 70 kg/year today [3]. Although the various national and international health agencies allow/recommend certain daily quantities of “added” salt and sugar, it must be emphasized that the addition of salt and sugar in the diet is not necessary as, except in very special cases, the sodium naturally contained in food would be sufficient for a correct diet, and the supply of glucose through complex carbohydrates would be the most correct way to take this nutrient.

The term “salt” unequivocally refers to sodium chloride, the main source of sodium intake, in addition to the less significant amount of sodium naturally contained in food. On the other hand, when we talk about “sugar”, some specifications are necessary. From a chemical point of view, the “simple sugars” are monosaccharides (glucose, fructose, and galactose) and disaccharides, which derive from combinations of monosaccharides, such as sucrose (glucose + fructose), lactose (glucose + galactose), and maltose (glucose + glucose). From a nutritional point of view, things are more complex. In fact, the sugars naturally contained in some foods such as lactose in milk and fructose in fruit (intrinsic sugars) must be considered distinct from those added in the production/preparation of foods (extrinsic sugars). To indicate extrinsic sugars, the definition of “added sugars” has been proposed, which in any case is not entirely satisfactory, since the sugars contained in honey, fruit juices, and nectars, theoretically intrinsic, are consumed in practice as if they were extrinsic. The WHO has proposed the definition of “free sugars”, and it does not consider lactose and galactose contained exclusively in milk as free sugars. On the other hand, the WHO considers glucose, fructose, and sucrose as free sugars only if added to food preparations, but not if taken directly with fruits. Furthermore, the saccharides contained in honey, fruit juices, or nectars are always considered “free sugars” [4,5,6,7,8,9]. The definition excludes small saccharide molecules (fructans, galactans, lactulose, and polyols) which are only minimally absorbed in the intestine and are not considered in this review. The largest share of sugar is introduced in the diet as sucrose (the common table sugar) and high-fructose sweetening syrups (HFCS) used widely, but not exclusively, in soft drinks. These syrups contain 55–60% fructose and are produced through the isomerization of the glucose contained in corn starch. In children, the sugars in honey and fruit juices may also have a certain quantitative importance, while the use of fructose alone, erroneously perceived as a natural sweetener, is becoming increasingly popular. We do not talk in detail about non-calorie sweeteners, which should still be viewed with suspicion, especially in children. Their use, moreover, does not seem to improve incorrect eating habits [10,11,12].

The purpose of this review is to explore the relationship between an excessive dietary intake of salt and sugar and the onset of arterial hypertension in children. The prevalence of excess weight in children has increased exponentially in recent decades, resulting in a modification of the epidemiology of pediatric hypertension. A recent meta-analysis reported an increment in the prevalence of hypertension from 2.4–3.0% to 4.3–5.3% in children between 6 and 11 years and from 4.3–5.3% to 6.1–7.9% in adolescents between 12 and 16 years, from 2000 to 2015 [13]. At the same time, the frequency of secondary hypertension in pediatric referral series decreased over time from 85% to 9% [14]. The prevalence of secondary hypertension is higher in younger children than in older children and adolescents, particularly when considering patient populations referred to tertiary pediatric hypertension clinics [15,16].

## 2. Recommendations for Dietary Salt and Sugar Intake in Children

The daily dietary intakes of sodium and salt recommended by health agencies for children are summarized in Table 1. As can be seen from the table, all the references substantially overlap. Under 6 months of age, no indications are given regarding the intake of sodium, as breast milk is considered the gold standard for all nutrients and, therefore, also for sodium. The sodium content of breast milk differs according to the mother’s geographical origin and her weight status, suggesting an important role in maternal eating habits [17]. However, it must be emphasized that the sodium content in breast milk is low (17 mg/100 mL) [18] and, based on this amount, breastmilk substitutes have been created, whereas cow’s milk has a sodium content about three times that of human milk. Even for this reason, its use is not recommended in infants. 

Data from the National Health and Nutrition Examination Survey (NHANES) 2003–2010 showed that more than 80% of children aged 1–5 years exceeded their sodium upper intake levels [19]. An Italian study provided evidence that, in a large sample of children aged 6–36 months, the intake of sodium and free carbohydrates was high. In particular, most of the children aged ≥12 months were taking more sodium than the daily upper limit of 1000 mg suggested by the Italian guidelines [20]. In older Italian children, the estimated intake of salt was about 7.0 g per day, which means that they had a consumption higher than the recommended standard dietary target [21].

Table 2 shows the recommendations regarding the maximum daily intake of free sugars. In this case, there is also consensus on a quantity that should not exceed 10% of the total caloric amount provided by the diet. Studies on fructose intake in the general population are few. The most significant data are provided by the NHANES, relating to the population of the United States in the years 1999–2004, which shows that the average daily consumption of fructose per capita was 49 g (of which only 8 g was provided by fruits). In the younger population, however, the intake was much higher (75 g among males aged 15 to 22) [22]. Moreover, in the Zuccotti study, very few babies had an intake of free sugar within the guidelines’ recommended value [20].

## 3. Salt and Pathogenesis of Arterial Hypertension

**Figure 1:**
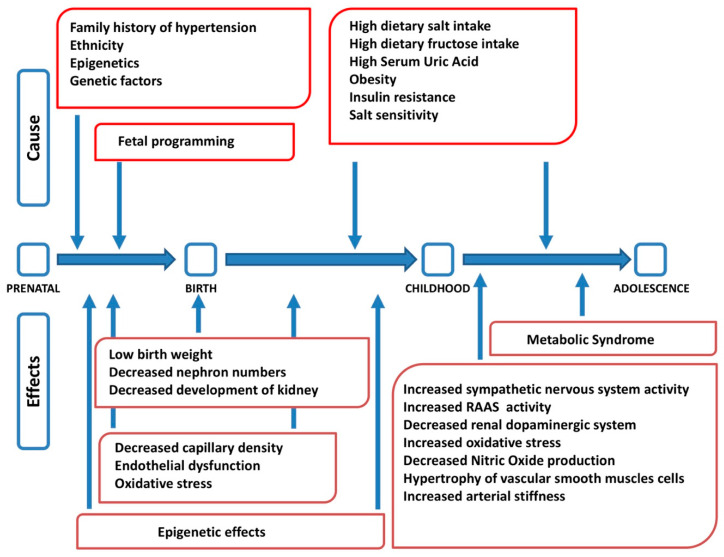
Factors interacting in the pathogenesis of arterial hypertension at different stages of life (modified from [23]). RAAS: renin angiotensin aldosterone system.

Arterial hypertension is a complex disease at the origin of which genetic, epigenetic, environmental, and behavioral factors interact, with different roles in different age stages (Figure 1) [23]. The etiopathogenesis of arterial hypertension, therefore, has a multifactorial origin [24]. Several studies have shown a correlation among dietary salt intake, blood pressure values, and morbidity and mortality from cardiovascular disease. It has also been shown that, regardless of body weight, gender, and age, salt intake is considered a well-established risk factor for hypertension [25]. However, the exact etiopathogenetic mechanisms that lead to the development of arterial hypertension and its relationship with an excessive intake of salt have not yet been fully clarified. Hypertension may occur if the pressure natriuresis mechanism is impaired, and changes in blood pressure with different salt dietary intake vary from individual to individual. Salt sensitivity is defined as a difference in mean arterial pressure between a low- and high-sodium diet > 10% [26]. Recently, Bigazzi et al. showed that genetic polymorphisms previously implicated in salt-sensitive hypertension in adults had an impact on blood pressure values and sodium excretion in a population of adolescents, suggesting the possibility of an impairment of the physiological systems of sodium handling at a young age already [27].

For a long time, it was thought that the main link between sodium and arterial hypertension was the expansion of the extracellular volume due to the osmotic effect of sodium, especially in salt-sensitive subjects. However, this mechanism has been questioned by the discovery that sodium can be stored in the body in a non-osmolar form [28]. More recently, it has been hypothesized that salt may exert its effect on blood pressure via different and more complex mechanisms. Excessive salt intake would cause alterations in the physiological systems that regulate cardiac, vascular, and/or renal function. In the presence of hypertension, particularly in obese subjects, there is an increase in sympathetic nervous system (SNS) activity [29]. This autonomic alteration is already present at pediatric age; in fact, greater sympathetic modulation and lower parasympathetic modulation have been described both in hypertensive children [30] and in adolescents [31]. Activation of the SNS, on the one hand, leads to an increase in sodium reabsorption at the level of the proximal convoluted tubule and, on the other hand, stimulates the renin angiotensin aldosterone system (RAAS) which, in turn, increases both distal renal sodium reabsorption and sympathetic activity via angiotensin II. In addition, it seems that various genetic, hormonal, and neuroendocrine factors are involved in the development of sodium-sensitive arterial hypertension [32]. The SNS, RAAS, natriuretic peptides, insulin, leptin, and other endothelial mediators with endocrine activity could all influence the sensitivity of blood pressure to salt intake [33]. 

The etiopathogenesis of arterial hypertension begins even before birth through an epigenetic mechanism called “fetal programming”, through the direct and/or indirect action of the salt introduced with the mother’s diet [34]. It has been shown in animal models that the intake of salt during the early stages of life could have a “programming” effect on blood pressure [35,36]. In other words, early salt exposure could lead to a permanent increase in blood pressure, even if salt intake were to decrease later in life. Moreover, studies conducted on rats have shown that a diet rich in salt during gestation and lactation results in an increase in blood pressure in males once they reach adulthood [37]. It has been hypothesized that, even in humans, “prenatal insults”, including excessive salt intake, can limit capillary density, endothelial function, and the maturation of the nephronic mass, thus favoring the onset of hypertension already in adolescents and in young adults [38].

Suckling et al. have suggested that the consumption of foods that are high in sodium leads to a transient increase in plasma sodium concentration which could have toxic effects on the vascular system [39]. Plasma sodium concentration may exert an effect on blood pressure by modifying the “stiffness” of endothelial cells. The greater “stiffness” of endothelial cells would lead to a reduction in the activity of nitric oxide synthase (eNOS) and an increase in vascular resistance, with a consequent increase in blood pressure [40]. This condition could, in turn, induce microvascular remodeling and a systemic proinflammatory state leading to microvascular endothelial inflammation, anatomical remodeling, and functional abnormalities, as shown in animal models [41]. The presence of a proinflammatory state involving the endothelium due to an excessive intake of salt has been demonstrated in several studies performed on rodents [42,43,44] and on humans [45,46]. These studies show that the negative effects of a high concentration of sodium on the vascular system are mediated by reactive oxygen species (predominantly superoxide, O_2_^−^). A high salt content in the diet results in an increase in O_2_^−^, which decreases the bioavailability of nitric oxide (NO), which is eliminated via transformation into its radicals. Therefore, a high sodium intake alters endothelial function through a reduced bioavailability of NO. In addition to increasing oxidative stress, a high sodium content can also decrease the antioxidant defense mechanisms by reducing the expression of superoxide dismutase [47,48,49].

## 4. Sugar and Pathogenesis of Arterial Hypertension

The free sugars in the diet are glucose, fructose, and sucrose. In the intestine, sucrose is broken down into its two components, glucose and fructose, which are absorbed as such. Therefore, to evaluate the consequences of sugar consumption on the development of arterial hypertension, both the effect of the total consumption of free sugars and the specific metabolic role of fructose compared to that of glucose must be considered. Furthermore, the relationship between blood pressure and uric acid, the final product of the catabolism of purines (adenosine and cytosine), a metabolic pathway differentiated from the metabolism of sugars, must also be considered. Foods that are rich in purines can lead to an increase in uricemia, but they hardly contain free sugars and, in general, are poorly consumed by children and adolescents. Excessive ingestion of fructose also leads to an increase in uricemia and, in children, is the main dietary cause of hyperuricemia. The effects on blood pressure and other parameters of the metabolic syndrome of free sugars considered as a whole, as well as those of fructose and uric acid, add up and enhance each other [50]. For clarity, all these aspects are dealt with separately.

### 4.1. Free Sugars

Free sugars, mainly consumed through the intake of sugary drinks (SSB), cause a high calorie intake in children [9,51,52]. In the United States, the calories provided by SSBs in children are equal to 7.3% of the daily caloric intake, with values of over 9% in adolescence [51]. Similar values have been found in English [53] and in Asian adolescents [54,55]. Sixteen percent of Italian adolescents say they take SSBs every day [56]. Free sugars, especially in liquid form, have a very low satiating effect and, therefore, do not limit the intake of calories from other sources, thus favoring the development of excess weight [57]. This has been demonstrated both in adolescents [58] and in children [59]. In particular, obesity appears to be the major risk factor for primary arterial hypertension in children [60,61].

### 4.2. Fructose

**Figure 2:**
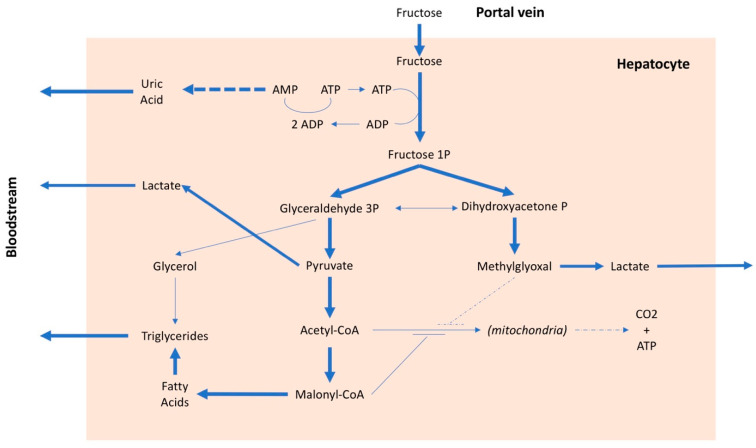
Metabolic pathways of fructose. AMP: adenosine monophosphate, ADP: adenosine diphosphate, ATP: adenosine triphosphate, CO2: carbon dioxide, CoA: coenzyme A, P: phosphate.

The metabolism of fructose differs from that of glucose. Glucose, after intestinal absorption, reaches the hepatocyte where it can have three different destinations: (i) to be stored as glycogen, (ii) to be secreted into the blood to be used as nourishment for the cells, mainly under insulin control, or (iii) to be split into two trioses (glyceraldehyde and hydroxyacetone), transformed first into pyruvate and then into acetyl-CoA and used by mitochondria to produce energy. In addition to being controlled by insulin, which stimulates its entry into cells and is the main regulator of plasma glucose levels, glucose is also regulated by internal systems of the hepatocyte, which are related to the energy levels present in the cell [62]. In the case of fructose, however, these internal systems do not work [63] and, therefore, fructose can enter the hepatocyte without any control. About 70–80% of the fructose present in the portal blood is absorbed at the first passage by the liver (the residual amount is metabolized by the kidney); consequently, the plasma quantity of fructose is minimal even after a meal with high fructose content [64]. The uncontrolled entry of fructose into the liver cells is the main cause of a series of metabolic alterations. The fructose in the hepatocyte is rapidly phosphorylated by phosphofructokinase and transformed into fructose 1 phosphate, with consumption of ATP (Figure 2). In the presence of a high amount of fructose, this entails, on the one hand, the lowering of the cell’s energy level and, on the other, due to the degradation of ATP, the production of adenosine and, subsequently, uric acid. Furthermore, an excessive absorption of fructose by the liver leads to the production of lactate, which favors the onset of insulin resistance, as well as an increase in the synthesis of fatty acids and cholesterol [65,66,67]. Therefore, after a high-fructose meal, there is an increase in uricemia [68,69] and very-low-density lipoprotein (VLDL) levels [70,71], while a certain amount of fatty acids remains in the adipocyte, promoting the development of nonalcoholic fatty liver disease [72]. A high fructose intake is, therefore, associated with an increase in free fatty acids, triglycerides, lactate, and methylglyoxal, all factors that can contribute to an increase in insulin resistance with a consequent increase in blood pressure values [73]. Furthermore, fructose, in addition to inducing insulin resistance, also determines resistance to leptin. Both conditions inhibit the center of satiety and stimulate food intake [74], favoring the onset of obesity and, consequently, arterial hypertension. It should be emphasized that insulin resistance is a factor independently associated with an increase in blood pressure values, even in children [68,75]. There is also an interaction between salt and fructose, because the latter may favor the reabsorption of sodium both in the kidney [76] and in the intestine [77], especially when associated with a high-salt diet [78]. Lastly, it has been suggested that continued consumption of fructose could lead to kidney damage [79] which, over time, could favor an increase in blood pressure.

### 4.3. Uric Acid

A large number of studies, both experimental and clinical, have shown an association between uric acid values and arterial hypertension in children [80,81,82]. The child is a “unique” clinical model for studying the mechanisms that lead to the development of primary hypertension, as, at this age, the role of confounding factors such as aging, long duration of hypertension, drug therapies, smoking, and other cardiovascular diseases are generally absent. The only clinical studies that demonstrated a cause/effect relationship between uric acid levels and blood pressure values were performed in pediatric age. Two randomized studies performed in hypertensive and prehypertensive adolescents showed a reduction in systolic and diastolic blood pressure values with the administration of uric acid-lowering drugs, suggesting a causal relationship between urate and hypertension in these subjects [82,83]. Moreover the antihypertensive efficacy of lifestyle modifications is blunted in children with high uric acid levels [84]. As already pointed out, the main cause of increased uricemia in children is excessive consumption of fructose, particularly through SSBs. Uric acid increases oxidative stress [85,86], as well as the production of tumor necrosis factor, interleukin 6, and other chemokines, stimulating inflammatory processes especially at the vascular level [87]. In addition, uric acid increases insulin resistance [88], favoring the onset of those alterations that characterize the metabolic syndrome, including arterial hypertension. However, the most important effects of uric acid in the development of hypertension are due to its direct action on arterioles and kidney. At the arteriolar level, uric acid inhibits the production of nitric oxide and the activity of endothelin, favoring the onset of endothelial dysfunction [89]. The result is an arteriolar vasoconstriction, which is reversible in the first phase but which, later, due to the proliferation of the arteriolar smooth muscle cells, becomes irreversible, with a stabilization of arterial hypertension. This progression of the hypertensive process suggests the need to perform interventions as early as possible, identifying the subjects at risk even in pediatric age [90]. Lastly, uric acid in the kidney may exert a nephrotoxic effect, which could contribute to the development of arterial hypertension [91].

## 5. Epidemiological Studies on Salt and Sugar Intake and Arterial Hypertension in Children

Several studies showed a correlation between high salt intake and increased blood pressure values and hypertension prevalence in the pediatric population. A recent meta-analysis showed that sodium intake is associated with blood pressure values in children and adolescents. Eighteen studies analyzing sodium intake and blood pressure values showed that, for every additional gram of sodium intake per day, systolic blood pressure (SBP) increased by 0.8 mmHg and diastolic blood pressure (DBP) by 0.7 mmHg. This association was stronger among children with excess weight and low potassium intake. Moreover, sodium reduction interventions decreased SBP and DBP by 0.6 mmHg and 1.2 mmHg, respectively [92].

In support of the hypothesis that high salt intake increases blood pressure in children, a meta-analysis performed several years ago that included 10 studies in children and adolescents demonstrated that a modest reduction in salt intake caused a decrease in blood pressure (1.17 and 1.29 mmHg of SBP and of DBP values, respectively), providing strong support for the importance of a reduction in salt dietary intake in pediatric age [35]. Since blood pressure tracks from childhood to adulthood, these findings suggest that a reduction in sodium intake during childhood and adolescence could lower blood pressure and prevent the development of hypertension later on in life.

Among the different methods available for assessing salt intake, sodium excretion over 24 h is currently the most accurate [93]. In boys aged 7 to 11 years, Apariciò et al. showed a correlation between SBP and DBP values with sodium excretion/24 h, with an increase of 0.04 mmHg for each mmol of Na excreted in 24 h [94]. Salt intake and excretion are higher in adolescents than in children. In a cohort study of 6- to 16-year-olds, Shi et al. found that urinary sodium excretion in 6-year-olds averaged 83 mmol Na/day and that this value increased to 100 mmol Na/day at 16 years of age. In this study, a 1 g/day increase in salt intake was associated with a 0.2 mmHg increase in SBP [95].

Correra-Costa et al. confirmed that an increase in salt consumption leads to an increase in daytime SBP and DBP, assessed by 24 h pressure monitoring, and suggested a gender difference in the effect of salt intake on SBP. In a population of children (age 8–9 years), there was in fact an association between salt intake in boys that was not observed in girls [96]. The amount of salt intake in children and adolescents depends on the family’s diet. It has been shown that sodium excretion of children is correlated to that of their parents [97,98]. Cotter pointed out that 91% of children consume amounts of salt similar to that taken by their parents, particularly that taken by their mother. In the children of mothers who add salt to the food after cooking, a higher sodium excretion was found, compared to the children of mothers who do not add it.

Few studies are available on the effects of dietary sodium intake on blood pressure in the first months of life. He’s meta-analysis, which also included a subgroup of three trials performed in infants, showed that a reduction in salt intake led to a significant reduction in SBP of 2.47 mm Hg [35]. The studies are quite old; as a matter of fact, research of this kind would currently not be considered ethical. Pomeranz et al. compared two groups of infants fed the same formula milk supplemented with two different amounts of sodium (32 mg/dL and 196 mg/dL) [99]. During the 2 months of follow-up, the group that assumed a higher sodium intake showed significantly higher SBP and DBP values. Another study [100] compared two groups of children, one assigned to a low-sodium diet and the other to a normal-sodium diet in the first 6 months of life. During these 6 months of follow-up, the high-sodium-intake group showed higher SBP values than the other group, and the difference increased over time. At the end of the study, SBP values of children fed a high-sodium diet exceeded those of the moderate intake group by 2.1 mmHg, an important difference for the age group considered. It is extremely interesting that, after 15 years, one-third of the study participants were re-evaluated, and the researchers found that the subjects who had a higher sodium intake in the first 6 months of life had higher SBP and DBP values (3.6 and 2.2 mmHg, respectively) [101].

Not only salt intake, but also high sugar intake is related to an increase in blood pressure values in children. Evidence has been reported in the literature about a correlation of blood pressure with both fructose consumption (especially taken with SSBs) [102] and serum uric acid values [81,103]. Since a high intake of fructose is associated with higher uric acid levels [91,104], in clinical studies, it is often difficult to separate the effects of uric acid from those of fructose on blood pressure. In a recent meta-analysis, which included 14 studies performed in children and adolescents, high consumption of SSBs was associated with an increase of 1.67 mmHg in SBP. Important consumers of SSBs were also 1.36 times more likely to develop hypertension than more modest users [103].

Studies that associate high serum uric acid values with the presence of hypertension in children are numerous. Furthermore, an association between hyperuricemia and incident hypertension has been demonstrated. It has been suggested that increases in uricemia in childhood may predict the onset of hypertension in adulthood [105]. In a meta-analysis that included 18 prospective studies, the presence of hyperuricemia was associated with a 40% increased risk of incident hypertension; for a 1 mg/dL increase in serum uric acid levels, the risk of developing hypertension was increased by 13%, and studies performed in older populations showed a lower risk than those performed in younger populations [106]. A systematic analysis of all trials related to uric acid and hypertension is beyond the scope of this review. However, it is important to point out that it has been described that, in a large, nationally representative cohort of healthy US adolescents with a low prevalence of cardiovascular disease and cardiovascular disease risk, for each 0.1 mg/dL increment in serum uric acid, there was a 1.38-fold increased risk of hypertension [107]. In addition, Viazzi et al. showed that, in a pediatric population at relatively high cardiovascular risk, the prevalence of arterial hypertension increased by at least 50% for each 1 mg/dL of serum uric acid increment and that children with serum uric acid values in the highest quartile had a risk of being hypertensive that was twice the risk observed in the lowest quartile (odds ratio (OR) = 2.04) [81]. In an important study, Nguyen et al. highlighted the link among SSBs, uric acid, and blood pressure. In a group of adolescents aged 12 to 18 years, serum uric acid levels were significantly associated with SSB intake (0.18 mg/dL from the lowest to the highest category of SSB consumption) and, in turn, SBP also increased with increasing SSB consumption (0.17 *z*-scores from the lowest to the highest category) [104].

## 6. Relationship between Salt and Sugar Intake and Intervention Programs to Reduce Salt and Sugar Consumption

It is generally thought that salt and sugar are taken by children through different foods and separately. Instead, the preparation of many desserts also includes the addition of salt to improve their palatability. Furthermore, current eating habits (Western diet) are characterized by a large consumption of both salt and sugar [108]. He et al. made a very important observation, establishing the presence of a strong association, which remained significant after adjustment for potential confounders, among salt, fluid, and SSB intake in a population of children and adolescents in Great Britain [109]. A difference of 1 g/day in salt consumption was associated with a difference of 100 and 27 g/day in total fluid and SSB intake, respectively. These findings suggest that, if salt intake in children is reduced, there may also be a reduction in SSB consumption and this could have a beneficial effect on both body weight and arterial pressure, regardless of the effect directly exerted by the low-sodium diet on blood pressure values [109].

The demonstrated correlation between salt and sugar intake in children can be explained not only by the natural and innate propensity for these flavors, but also by the eating habits of the parents [110]. Lastly, an excessive sodium intake, creating a transient hyperosmolarity at the level of the portal vein and liver, activates the polyol pathway determining the endogenous production of fructose [111,112] which, adding to the amount of fructose taken with the diet, can promote the development of metabolic syndrome. For these reasons, the prevention and dietary treatment of arterial hypertension at a pediatric age, as well as the reduction of excess weight if present, should be based on the reduction of the consumption of salt and sugar, particularly fructose [113]. In a cohort of overweight and/or hypertensive children, these measures proved effective and resulted in a significant reduction in blood pressure, regardless of the presence of arterial hypertension [114]. 

Intervention programs to reduce salt and sugar consumption have been proposed, especially aimed at populations with high cardiovascular risk [115]. Within these programs, special interventions for industries are also envisaged, to encourage the production of foods with healthier nutritional characteristics, which could also benefit people who are not at high cardiovascular risk, particularly children. An example of this type of intervention is the Italian project for the production of bread with a reduced amount of salt, which is part of the European initiatives for the reduction of dietary sodium intake [116]. With regard to the pediatric age, the intervention projects mainly aim at reducing the childhood obesity through correct eating habits and lifestyles [117]. All intervention programs designed for children include indications to increase the consumption of fruit and vegetables, to reduce the intake of free sugars, and to stimulate physical activity [50]. Nutritional education programs are mainly offered in schools, which constitute microenvironments in which children spend a large part of their time and which play a pivotal role in influencing their choices. In this way, a collaboration can be created among families, educators, and policymakers [117]. Intervention strategies in schools should involve the managers of school catering services, in order to offer fresh food, quality products, and well-balanced meals from a nutritional point of view. In this way, opportunities can be created to introduce notions about nutritional education to children and families. Another important point would be to succeed in banning food vending machines in schools [118]. It has been shown that school nutritional education projects, when implemented, lead to a reduction in the consumption of sweet drinks [119], soda drinks [120], and SSBs [121]. Some countries have also imposed taxes on soft drinks sold in grocery stores and vending machines, thus trying to decrease calorie intake from nutrient-poor foods. However, these taxes have often not been effective in reducing the consumption of sweet drinks [122]. To date, no interventions have been planned that consider the evidence of the role of fructose consumption in the development of metabolic syndrome. Informative interventions on this topic would be appropriate, as the consumption of added fructose is generally perceived as healthy by the general population.

## 7. Conclusions

Cardiovascular diseases occur in adulthood, but the underlying vascular alterations begin very early in childhood and are related to the presence of risk factors such as arterial hypertension and obesity. Salt and sugar, if taken in excess, are important risk factors for hypertension and obesity. In consideration of the importance of early prevention, both at the individual level and at the level of public health, it would be mandatory to apply strategies for limiting the consumption of salt and free sugars, particularly fructose, in children.

## Figures and Tables

**Table 1:** Recommended intake of sodium and salt in children and adolescents.

WHO	DRI		NHS		LARN		
The amount of 2 g of Na (5 g NaCl) recommended in adults, reduced proportionally to dietary calories	Age	AI g Na (NaCl)	Age	AI g Na (NaCl)	Age	AI g Na (NaCl)	SDT g Na (NaCl)
	6–12 months	0.37 (0.9)	6–12 months	n.d.	6–12 months	0.4 (1.0)	n.d.
	1–3 years	0–8 (2.0)	1–3 years	0.8 (2.0)	1–3 years	0.7 (1.75)	0.9 (2.25)
	4–8 years	1 (2.5)	4–6 years	1.2 (3.0)	4–6 years	0.9 (2.25)	1.2 (3.0)
	9–13 years	1.2 (3.0)	7–10 years	2 (5.0)	7–10 years	1.1 (2.75)	1.5 (3.75)
	14–18 years	1.5 (3.75)	>11 years	2.4 (6.0)	>11 years	1.5 (3.75)	2.0 (5.0)

WHO: World Health Organization, DRI: Dietary Reference Intake of United States, NHS: National Health Service of United Kingdom, LARN: intake recommended in Italy, AI: adequate intake, SDT: recommended intake for the prevention of noncommunicable diseases, n.d.: not defined.

**Table 2:** Recommended intake of sugar in pediatric age.

WHO	<10% of dietary calories
ESPGHAN	<5% of dietary calories
LARN	<15% of dietary calories (including fruit sugars)

WHO: World Health Organization, LARN: intake recommended in Italy, ESPGHAN: European Society for Pediatric Gastroenterology, Hepatology, and Nutrition.

## Data Availability

Not applicable.

## References

[1] Mennella J.A. (2014). Ontogeny of taste preferences: Basic biology and implications for health. Am. J. Clin. Nutr..

[2] Mozaffarian D., Fahimi S., Singh G.M., Micha R., Khatibzadeh S., Engell R.E., Lim S., Danaei G., Ezzati M., Powles J. (2014). Global Sodium Consumption and Death from Cardiovascular Causes. N. Engl. J. Med..

[3] Johnson R.J., Segal M.S., Sautin Y., Nakagawa T., Feig D.I., Kang D.-H., Gersch M.S., Benner S., Sánchez-Lozada L.G. (2007). Potential role of sugar (fructose) in the epidemic of hypertension, obesity and the metabolic syndrome, diabetes, kidney disease, and cardiovascular disease. Am. J. Clin. Nutr..

[4] Vos M.B., Kaar J.L., Welsh J.A., Van Horn L.V., Feig D.I., Anderson C.A., Patel M.J., Munos J.C., Krebs N.F., Xanthakos S.A. (2017). Added Sugars and Cardiovascular Disease Risk in Children: A Scientific Statement from the American Heart Association. Circulation.

[5] WHO (2018). Organization World Health Guideline: Sugars Intake for Adults and Children.

[6] Scientific Advisory Committee on Nutrition (2015). Carbohydrates and Health.

[7] Bresson J., Flynn A., Heinonen M., Hulshof K., Korhonen H., Lagiou P., Løvik M., Marchelli R., Martin A., Moseley B. (2009). Review of labelling reference intake values—Scientific Opinion of the Panel on Dietetic Products, Nutrition and Allergies on a request from the Commission related to the review of labelling reference intake values for selected nutritional elements. EFSA J..

[8] Institute of Medicine of the National Academies (2005). Dietary Reference Intakes for Energy, Carbohydrate, Fiber, Fat, Fatty Acids, Cholesterol, Protein, and Amino Acids.

[9] Mis N.F., Braegger C., Bronsky J., Campoy C., Domellöf M., Embleton N.D., Hojsak I., Hulst J., Indrio F., Lapillonne A. (2017). Sugar in Infants, Children and Adolescents. J. Pediatr. Gastroenterol. Nutr..

[10] Toews I., Lohner S., De Gaudry D.K., Sommer H., Meerpohl J.J. (2019). Association between intake of non-sugar sweeteners and health outcomes: Systematic review and meta-analyses of randomised and non-randomised controlled trials and observational studies. BMJ.

[11] Baker-Smith C.M., De Ferranti S.D., Cochran W.J. (2019). The Use of Nonnutritive Sweeteners in Children. Pediatrics.

[12] Pepino M.Y. (2015). Metabolic effects of non-nutritive sweeteners. Physiol. Behav..

[13] Song P., Zhang Y., Yu J., Zha M., Zhu Y., Rahimi K., Rudan I. (2019). Global Prevalence of Hypertension in Children. JAMA Pediatr..

[14] Flynn J. (2013). The changing face of pediatric hypertension in the era of the childhood obesity epidemic. Pediatr. Nephrol..

[15] Flynn J.T., Daniels S.R., Hayman L.L., Maahs D.M., McCrindle B.W., Mitsnefes M., Zachariah J.P., Urbina E.M. (2014). Update: Ambulatory Blood Pressure Monitoring in Children and Adolescents. Hypertension.

[16] Gupta-Malhotra M., Banker A., Shete S., Hashmi S.S., Tyson J.E., Barratt M.S., Hecht J.T., Milewicz D.M., Boerwinkle E. (2015). Essential Hypertension vs. Secondary Hypertension Among Children. Am. J. Hypertens..

[17] Sánchez C., Fente C., Barreiro R., López-Racamonde O., Cepeda A., Regal P. (2020). Association between Breast Milk Mineral Content and Maternal Adherence to Healthy Dietary Patterns in Spain: A Transversal Study. Foods.

[18] Koo W.W., Gupta J.M. (1982). Breast milk sodium. Arch. Dis. Child..

[19] Tian N., Zhang Z., Loustalot F., Yang Q., Cogswell M.E. (2013). Sodium and potassium intakes among US infants and preschool children, 2003–2010. Am. J. Clin. Nutr..

[20] Zuccotti G.V., Cassatella C., Morelli A., Cucugliato M.C., Catinello G., Del Balzo V., Guidarelli L., Agostoni C., Mameli C., Troiano E. (2014). Nutrient Intake in Italian Infants and Toddlers from North and South Italy: The Nutrintake 636 Study. Nutrients.

[21] Campanozzi A., Avallone S., Barbato A., Iacone R., Russo O., De Filippo G., D’Angelo G., Pensabene L., Malamisura B., Cecere G. (2015). High Sodium and Low Potassium Intake among Italian Children: Relationship with Age, Body Mass and Blood Pressure. PLoS ONE.

[22] Marriott B.P., Cole N., Lee E. (2009). National Estimates of Dietary Fructose Intake Increased from 1977 to 2004 in the United States. J. Nutr..

[23] Tiu A.C., Bishop M.D., Asico L.D., Jose P.A., Villar V.A.M. (2017). Primary Pediatric Hypertension: Current Understanding and Emerging Concepts. Curr. Hypertens. Rep..

[24] Nelson W.E., Kliegman R. (2010). Nelson’s Textbook of Pediatrics.

[25] Ekmekcioglu C., Blasche G., Dorner T.E. (2013). Too Much Salt and How We Can Get Rid of It. Forsch. Komplementarmed..

[26] Weinberger M.H. (1996). Salt Sensitivity of Blood Pressure in Humans. Hypertension.

[27] Bigazzi R., Zagato L., Lanzani C., Fontana S., Messaggio E., Carpini S.D., Citterio L., Simonini M., Brioni E., Magnaghi C. (2020). Hypertension in High School Students: Genetic and Environmental Factors. Hypertension.

[28] Laffer C.L., Scott R.C., Titze J.M., Luft F.C., Elijovich F. (2016). Hemodynamics and Salt-and-Water Balance Link Sodium Storage and Vascular Dysfunction in Salt-Sensitive Subjects. Hypertension.

[29] Grassi G. (2010). Sympathetic Neural Activity in Hypertension and Related Diseases. Am. J. Hypertens..

[30] Genovesi S., Pieruzzi F.U.E.G., Giussani M., Tono V., Stella A., Porta A., Pagani M., Lucini D. (2008). Analysis of Heart Period and Arterial Pressure Variability in Childhood Hypertension. Hypertension.

[31] Farah B.Q., Barros M.V., Balagopal B., Ritti-Dias R.M. (2014). Heart Rate Variability and Cardiovascular Risk Factors in Adolescent Boys. J. Pediatr..

[32] Galletti F., Strazzullo P. (2016). The blood pressure–salt sensitivity paradigm: Pathophysiologically sound yet of no practical value. Nephrol. Dial. Transplant..

[33] Elijovich F., Weinberger M.H., Anderson C.A., Appel L.J., Bursztyn M., Cook N.R., Dart R.A., Newton-Cheh C.H., Sacks F.M., Laffer C.L. (2016). Salt Sensitivity of Blood Pressure. Hypertension.

[34] Nuyt A.M., Alexander B.T. (2009). Developmental programming and hypertension. Curr. Opin. Nephrol. Hypertens..

[35] He F.J., MacGregor G.A. (2006). Importance of Salt in Determining Blood Pressure in Children. Hypertension.

[36] Dahl L.K., Kudsen K.D., Heine M.A. (1968). Effects of Chronic Excess Salt Ingestion. Modification of experimental hypertension in the rat by variations in the diet. Circulation.

[37] Tain Y.-L., Lee W.-C., Wu K.L., Leu S., Chan J.Y. (2016). Targeting arachidonic acid pathway to prevent programmed hypertension in maternal fructose-fed male adult rat offspring. J. Nutr. Biochem..

[38] Lopez-Lopez J., Lopez-Jaramillo P., Camacho P.A., Gomez-Arbelaez D., Cohen D.D. (2015). The Link between Fetal Programming, Inflammation, Muscular Strength, and Blood Pressure. Mediat. Inflamm..

[39] Suckling R.J., He F.J., Markandu N.D., MacGregor G.A. (2012). Dietary salt influences postprandial plasma sodium concentration and systolic blood pressure. Kidney Int..

[40] Oberleithner H., Riethmüller C., Schillers H., MacGregor G.A., De Wardener H.E., Hausberg M. (2007). Plasma sodium stiffens vascular endothelium and reduces nitric oxide release. Proc. Natl. Acad. Sci. USA.

[41] Marketou M.E., Maragkoudakis S., Anastasiou I., Nakou H., Plataki M., Vardas P.E., Parthenakis F.I. (2019). Salt-induced effects on microvascular function: A critical factor in hypertension mediated organ damage. J. Clin. Hypertens..

[42] Lenda D.M., Sauls B.A., Boegehold M.A. (2000). Reactive oxygen species may contribute to reduced endothelium-dependent dilation in rats fed high salt. Am. J. Physiol. Circ. Physiol..

[43] Lenda D.M., Boegehold M.A. (2002). Effect of a high-salt diet on oxidant enzyme activity in skeletal muscle microcirculation. Am. J. Physiol. Circ. Physiol..

[44] Guers J.J., Kasecky-Lardner L., Farquhar W.B., Edwards D.G., Lennon S.L. (2019). Voluntary wheel running prevents salt-induced endothelial dysfunction: Role of oxidative stress. J. Appl. Physiol..

[45] Greaney J.L., Dupont J.J., Lennon-Edwards S.L., Sanders P.W., Edwards D.G., Farquhar W.B. (2012). Dietary sodium loading impairs microvascular function independent of blood pressure in humans: Role of oxidative stress. J. Physiol..

[46] Cavka A., Jukic I., Ali M., Goslawski M., Bian J.-T., Wang E., Drenjancevic I., Phillips S.A. (2016). Short-term high salt intake reduces brachial artery and microvascular function in the absence of changes in blood pressure. J. Hypertens..

[47] Sessa W.C. (2004). eNOS at a glance. J. Cell Sci..

[48] Wray D.W., Witman M.A.H., Ives S.J., Mcdaniel J., Trinity J.D., Conklin J.D., Supiano M.A., Richardson R.S. (2013). Does Brachial artery FMD provide a bioassay for Nitric Oxide?. Hypertension.

[49] Loscalzo J. (2013). The Identification of Nitric Oxide as Endothelium-Derived Relaxing Factor. Circ. Res..

[50] Orlando A., Cazzaniga E., Giussani M., Palestini P., Genovesi S. (2018). Hypertension in Children: Role of Obesity, Simple Carbohydrates, and Uric Acid. Front. Public Health.

[51] Rosinger A., Herrick K., Gahche J., Park S. (2017). Sugar-sweetened Beverage Consumption Among U.S. Youth, 2011–2014. NCHS Data Brief.

[52] Bray G.A. (2013). Energy and Fructose from Beverages Sweetened with Sugar or High-Fructose Corn Syrup Pose a Health Risk for Some People. Adv. Nutr..

[53] Ng S.W., Ni Mhurchu C., Jebb S.A., Popkin B.M. (2011). Patterns and trends of beverage consumption among children and adults in Great Britain, 1986–2009. Br. J. Nutr..

[54] Han E., Kim T.H., Powell L.M. (2013). Beverage consumption and individual-level associations in South Korea. BMC Public Health.

[55] Lin W.-T., Huang H.-L., Huang M.-C., Chan T.-F., Ciou S.-Y., Lee C.-Y., Chiu Y.-W., Duh T.-H., Lin P.-L., Wang T.-N. (2012). Effects on uric acid, body mass index and blood pressure in adolescents of consuming beverages sweetened with high-fructose corn syrup. Int. J. Obes..

[56] Salute e Benessere Degli Adolescenti: Il Rapporto Internazionale con i Dati 2017/2018 HBSC. https://www.epicentro.iss.it/hbsc/rapporto-internazionale2017-2018.

[57] Malik V.S., Schulze M.B., Hu F.B. (2006). Intake of sugar-sweetened beverages and weight gain: A systematic review1–3. Am. J. Clin. Nutr..

[58] Ebbeling C.B., Feldman H.A., Chomitz V.R., Antonelli T.A., Gortmaker S.L., Osganian S.K., Ludwig D.S. (2012). A Randomized Trial of Sugar-Sweetened Beverages and Adolescent Body Weight. N. Engl. J. Med..

[59] De Ruyter J.C., Olthof M.R., Seidell J.C., Katan M.B. (2012). A Trial of Sugar-free or Sugar-Sweetened Beverages and Body Weight in Children. N. Engl. J. Med..

[60] Genovesi S., Giussani M., Pieruzzi F., Vigorita F., Arcovio C., Cavuto S., Stella A. (2005). Results of blood pressure screening in a population of school-aged children in the province of Milan: Role of overweight. J. Hypertens..

[61] Genovesi S., Antolini L., Giussani M., Brambilla P., Barbieri V., Galbiati S., Mastriani S., Sala V., Valsecchi M.G., Stella A. (2010). Hypertension, Prehypertension, and Transient Elevated Blood Pressure in Children: Association with Weight Excess and Waist Circumference. Am. J. Hypertens..

[62] Gugliucci A. (2019). Fructose at the crossroads of the metabolic syndrome and obesity epidemics. Front. Biosci..

[63] Tappy L., Lê K.-A. (2010). Metabolic Effects of Fructose and the Worldwide Increase in Obesity. Physiol. Rev..

[64] Petersen A., Kappler F., Szwergold B.S., Brown T.R. (1992). Fructose metabolism in the human erythrocyte. Phosphorylation to fructose 3-phosphate. Biochem. J..

[65] Zhang D.-M., Jiao R.-Q., Kong L.-D. (2017). High Dietary Fructose: Direct or Indirect Dangerous Factors Disturbing Tissue and Organ Functions. Nutrition.

[66] Gugliucci A. (2016). Fructose surges damage hepatic adenosyl-monophosphate-dependent kinase and lead to increased lipogenesis and hepatic insulin resistance. Med. Hypotheses.

[67] Russo E., Leoncini G., Esposito P., Garibotto G., Pontremoli R., Viazzi F. (2020). Fructose and Uric Acid: Major Mediators of Cardiovascular Disease Risk Starting at Pediatric Age. Int. J. Mol. Sci..

[68] Madero M., Perez-Pozo S.E., Jalal D., Johnson R.J., Sanchez-Lozada L.-G. (2010). Dietary Fructose and Hypertension. Curr. Hypertens. Rep..

[69] Nakagawa T., Hu H., Zharikov S., Tuttle K.R., Short R.A., Glushakova O., Ouyang X., Feig D.I., Block E.R., Herrera-Acosta J. (2006). A causal role for uric acid in fructose-induced metabolic syndrome. Am. J. Physiol. Physiol..

[70] Stanhope K.L., Bremer A.A., Medici V., Nakajima K., Ito Y., Nakano T., Chen G., Fong T.H., Lee V., Menorca R.I. (2011). Consumption of Fructose and High Fructose Corn Syrup Increase Postprandial Triglycerides, LDL-Cholesterol, and Apolipoprotein-B in Young Men and Women. J. Clin. Endocrinol. Metab..

[71] Akram M., Hamid A. (2013). Mini review on fructose metabolism. Obes. Res. Clin. Pr..

[72] Jin R., Willment A., Patel S.S., Sun X., Song M., Mannery Y.O., Kosters A., McClain C.J., Vos M.B. (2014). Fructose Induced Endotoxemia in Pediatric Nonalcoholic Fatty Liver Disease. Int. J. Hepatol..

[73] Johnson R.J., Stenvinkel P., Andrews P., Sánchez-Lozada L.G., Nakagawa T., Gaucher E., Andres-Hernando A., Rodriguez-Iturbe B., Jimenez C.R., Garcia G. (2020). Fructose metabolism as a common evolutionary pathway of survival associated with climate change, food shortage and droughts. J. Intern. Med..

[74] Shapiro A., Mu W., Roncal C., Cheng K.-Y., Johnson R.J., Scarpace P.J. (2008). Fructose-induced leptin resistance exacerbates weight gain in response to subsequent high-fat feeding. Am. J. Physiol. Integr. Comp. Physiol..

[75] Genovesi S., Brambilla P., Giussani M., Galbiati S., Mastriani S., Pieruzzi F., Stella A., Valsecchi M.G., Antolini L. (2012). Insulin resistance, prehypertension, hypertension and blood pressure values in paediatric age. J. Hypertens..

[76] Jayalath V.H., De Souza R.J., Ha V., Mirrahimi A., Blanco-Mejia S., Di Buono M., Jenkins A.L., Leiter L.A., Wolever T.M.S., Beyene J. (2015). Sugar-sweetened beverage consumption and incident hypertension: A systematic review and meta-analysis of prospective cohorts. Am. J. Clin. Nutr..

[77] Gordish K.L., Kassem K.M., Ortiz P.A., Beierwaltes W.H. (2017). Moderate (20%) fructose-enriched diet stimulates salt-sensitive hypertension with increased salt retention and decreased renal nitric oxide. Physiol. Rep..

[78] Cirillo P., Gersch M.S., Mu W., Scherer P.M., Kim K.M., Gesualdo L., Henderson G.N., Johnson R.J., Sautin Y.Y. (2009). Ketohexokinase-Dependent Metabolism of Fructose Induces Proinflammatory Mediators in Proximal Tubular Cells. J. Am. Soc. Nephrol..

[79] Fang J., Alderman M.H. (2000). Serum Uric Acid and Cardiovascular Mortality the NHANES I epidemiologic follow-up study, 1971-1992. National Health and Nutrition Examination Survey. JAMA.

[80] Feig D.I., Madero M., Jalal D.I., Sanchez-Lozada L.G., Johnson R.J. (2013). Uric Acid and the Origins of Hypertension. J. Pediatr..

[81] Viazzi F., Antolini L., Giussani M., Brambilla P., Galbiati S., Mastriani S., Stella A., Pontremoli R., Valsecchi M.G., Genovesi S. (2013). Serum Uric Acid and Blood Pressure in Children at Cardiovascular Risk. Pediatrics.

[82] Feig D.I., Soletsky B., Johnson R.J. (2008). Effect of Allopurinol on Blood Pressure of Adolescents with Newly Diagnosed Essential Hypertension. JAMA.

[83] Soletsky B., Feig D.I. (2012). Uric Acid Reduction Rectifies Prehypertension in Obese Adolescents. Hypertension.

[84] Lanaspa M.A., Sanchez-Lozada L.G., Choi Y.-J., Cicerchi C., Kanbay M., Roncal-Jimenez C.A., Ishimoto T., Li N., Marek G., Duranay M. (2012). Uric Acid Induces Hepatic Steatosis by Generation of Mitochondrial Oxidative Stress: Potential Role in Fructose-Dependent and- Independent Fatty Liver. J. Biol. Chem..

[85] Sautin Y.Y., Nakagawa T., Zharikov S., Johnson R.J. (2007). Adverse effects of the classic antioxidant uric acid in adipocytes: NADPH oxidase-mediated oxidative/nitrosative stress. Am. J. Physiol. Physiol..

[86] Chen L., Lan Z., Lin Q., Mi X., He Y., Wei L., Lin Y., Zhang Y., Deng X. (2013). Polydatin ameliorates renal injury by attenuating oxidative stress-related inflammatory responses in fructose-induced urate nephropathic mice. Food Chem. Toxicol..

[87] Kanbay M., Segal M., Afsar B., Kang D.-H., Rodriguez-Iturbe B., Johnson R.J. (2013). The role of uric acid in the pathogenesis of human cardiovascular disease. Heart.

[88] Choi Y., Yoon Y., Lee K., Hien T.T., Kang K.W., Kim K., Lee J., Lee M., Lee S.M., Kang D. (2014). Uric acid induces endothelial dysfunction by vascular insulin resistance associated with the impairment of nitric oxide synthesis. FASEB J..

[89] Su H.-Y., Yang C., Liang D., Liu H.-F. (2020). Research Advances in the Mechanisms of Hyperuricemia-Induced Renal Injury. BioMed. Res. Int..

[90] Strambi M., Giussani M., Ambruzzi M.A., Brambilla P., Corrado C., Giordano U., Maffeis C., Maringhin S., Matteucci M.C., Menghetti E. (2016). Novelty in hypertension in children and adolescents: Focus on hypertension during the first year of life, use and interpretation of ambulatory blood pressure monitoring, role of physical activity in prevention and treatment, simple carbohydrates and uric acid as risk factors. Ital. J. Pediatr..

[91] Kelishadi R., Mansourian M., Heidari-Beni M. (2014). Association of fructose consumption and components of metabolic syndrome in human studies: A systematic review and meta-analysis. Nutrition.

[92] Leyvraz M., Chatelan A., Da Costa B.R., Taffé P., Paradis G., Bovet P., Bochud M., Chiolero A. (2018). Sodium intake and blood pressure in children and adolescents: A systematic review and meta-analysis of experimental and observational studies. Int. J. Epidemiol..

[93] WHO (2012). WHO Guideline: Sodium Intake for Adults and Children.

[94] Aparicio A., Rodríguez-Rodríguez E., Cuadrado-Soto E., Navia B., López-Sobaler A.M., Ortega R.M. (2015). Estimation of salt intake assessed by urinary excretion of sodium over 24 h in Spanish subjects aged 7–11 years. Eur. J. Nutr..

[95] Shi L., Krupp D., Remer T. (2013). Salt, fruit and vegetable consumption and blood pressure development: A longitudinal investigation in healthy children. Br. J. Nutr..

[96] Correia-Costa L., Cosme D., Nogueira-Silva L., Morato M., Sousa T., Moura C., Mota C., Guerra A., Albino-Teixeira A., Areias J.C. (2015). Gender and obesity modify the impact of salt intake on blood pressure in children. Pediatr. Nephrol..

[97] Cotter J., Cotter M.J., Oliveira P., Cunha P., Torres E., Polonia J. (2019). Comparison of Salt Intake in Children to that of their Parents. Nephron.

[98] Cuadrado-Soto E., Peral-Suarez Á., Rodríguez-Rodríguez E., Aparicio A., Andrés P., Ortega R.M., López-Sobaler A.M. (2019). The association of parents’ behaviors related to salt with 24 h urinary sodium excretion of their children: A Spanish cross-sectional study. PLoS ONE.

[99] Pomeranz A., Dolfin T., Korzets Z., Eliakim A., Wolach B. (2002). Increased sodium concentrations in drinking water increase blood pressure in neonates. J. Hypertens..

[100] Hofman A., Hazebroek A., Valkenburg H.A. (1983). A Randomized Trial of Sodium Intake and Blood Pressure in Newborn Infants. JAMA.

[101] Geleijnse J.M., Hofman A., Witteman J.C., Hazebroek A.A., Valkenburg H.A., Grobbee D.E. (1997). Long-term Effects of Neonatal Sodium Restriction on Blood Pressure. Hypertension.

[102] Ha V., Jayalath V.H., Cozma A.I., Mirrahimi A., De Souza R.J., Sievenpiper J.L. (2013). Fructose-Containing Sugars, Blood Pressure, and Cardiometabolic Risk: A Critical Review. Curr. Hypertens. Rep..

[103] Feig D.I., Johnson R.J. (2003). Hyperuricemia in Childhood Primary Hypertension. Hypertension.

[104] Nguyen S., Choi H.K., Lustig R.H., Hsu C.-Y. (2009). Sugar-Sweetened Beverages, Serum Uric Acid, and Blood Pressure in Adolescents. J. Pediatr..

[105] Alper A.B., Chen W., Yau L., Srinivasan S.R., Berenson G.S., Hamm L.L. (2005). Childhood Uric Acid Predicts Adult Blood Pressure. Hypertension.

[106] Grayson P.C., Kim S.Y., LaValley M., Choi H.K. (2010). Hyperuricemia and incident hypertension: A systematic review and meta-analysis. Arthritis Rheum..

[107] Loeffler L.F., Navas-Acien A., Brady T.M., Miller E.R., Fadrowski J.J. (2012). Uric Acid Level and Elevated Blood Pressure in US Adolescents. Hypertension.

[108] Western Diet. https://medical-dictionary.thefreedictionary.com/Western+diet.

[109] He F.J., Marrero N.M., MacGregor G.A. (2008). Salt Intake Is Related to Soft Drink Consumption in Children and Adolescents. Hypertension.

[110] Mennella J.A., Finkbeiner S., Lipchock S.V., Hwang L.-D., Reed D.R. (2014). Preferences for Salty and Sweet Tastes Are Elevated and Related to Each Other during Childhood. PLoS ONE.

[111] Lanaspa M.A., Ishimoto T., Li N., Cicerchi C., Orlicky D.J., Ruzycki P., Rivard C.J., Inaba S., Roncal-Jimenez C.A., Bales E.S. (2013). Endogenous fructose production and metabolism in the liver contributes to the development of metabolic syndrome. Nat. Commun..

[112] Lanaspa M.A., Kuwabara M., Andres-Hernando A., Li N., Cicerchi C., Jensen T., Orlicky D.J., Roncal-Jimenez C.A., Ishimoto T., Nakagawa T. (2018). High salt intake causes leptin resistance and obesity in mice by stimulating endogenous fructose production and metabolism. Proc. Natl. Acad. Sci. USA.

[113] Spagnolo A., Giussani M., Ambruzzi A.M., Bianchetti M., Maringhini S., Matteucci M.C., Menghetti E., Salice P., Simionato L., Strambi M. (2013). Focus on prevention, diagnosis and treatment of hypertension in children and adolescents. Ital. J. Pediatr..

[114] Genovesi S., Orlando A., Rebora P., Giussani M., Antolini L., Nava E., Parati G., Valsecchi M.G. (2018). Effects of Lifestyle Modifications on Elevated Blood Pressure and Excess Weight in a Population of Italian Children and Adolescents. Am. J. Hypertens..

[115] Ponzo V., Pellegrini M., Costelli P., Laura V., Eusebio C.D., Ghigo E., Bo S. (2021). Strategies for Reducing Salt and Sugar Intakes in Individuals at Increased Cardiometabolic Risk. Nutrients.

[116] Strazzullo P., Campanozzi A., Avallone S. (2012). Does salt intake in the first two years of life affect the development of cardiovascular disorders in adulthood?. Nutr. Metab. Cardiovasc. Dis..

[117] Lobstein T., Baur L. (2005). Policies to prevent childhood obesity in the European Union. Eur. J. Public Health.

[118] Hercberg S., Chat-Yung S., Chauliac M. (2008). The French National Nutrition and Health Program: 2001–2006–2010. Int. J. Public Health.

[119] Taylor R.W., McAuley K.A., Barbezat W., Strong A., Williams S.M., Mann J.I. (2007). APPLE Project: 2-y findings of a community-based obesity prevention program in primary school–age children. Am. J. Clin. Nutr..

[120] James J., Thomas P., Cavan D., Kerr D. (2004). Preventing childhood obesity by reducing consumption of carbonated drinks: Cluster randomised controlled trial. BMJ.

[121] Rauba J., Tahir A., Milford B., Toll A., Benedict V., Wang C., Chehab L., Sanborn T. (2017). Reduction of Sugar-Sweetened Beverage Consumption in Elementary School Students Using an Educational Curriculum of Beverage Sugar Content. Glob. Pediatr. Health.

[122] Dilk A., Savaiano D.A. (2017). Sugar Price Supports and Taxation. Nutr. Today.

